# Effectiveness of a Primary Care Telerehabilitation Program for Post-COVID-19 Patients: A Feasibility Study

**DOI:** 10.3390/jcm10194428

**Published:** 2021-09-27

**Authors:** Marcelo Dalbosco-Salas, Rodrigo Torres-Castro, Andrés Rojas Leyton, Franco Morales Zapata, Elisabeth Henríquez Salazar, Gabriel Espinoza Bastías, María Elizabeth Beltrán Díaz, Kris Tapia Allers, Daniela Mornhinweg Fonseca, Jordi Vilaró

**Affiliations:** 1 Dirección de Salud de San Bernardo, Santiago 8070894, Chile; marcelo.dalbosco@gmail.com; 2 Escuela de Kinesiología, Facultad de Ciencias de la Salud, Universidad de las Américas, Santiago 7500975, Chile; 3 Department of Physical Therapy, Faculty of Medicine, University of Chile, Santiago 8380453, Chile; 4 International Physiotherapy Research Network (PhysioEvidence), 08025 Barcelona, Spain; jordi.gestos@gmail.com; 5 CESFAM Joan Alsina, San Bernardo, Santiago 8080429, Chile; andres.fernando20@gmail.com; 6 CESFAM Raúl Brañes, San Bernardo, Santiago 8051991, Chile; matias.mg2h@gmail.com; 7 CESFAM Carol Urzúa, San Bernardo, Santiago 8070175, Chile; elisabeth.kine@gmail.com; 8 CESFAM El Manzano, San Bernardo, Santiago 8051031, Chile; espinozabastiasgabriel@gmail.com; 9 CESFAM Confraternidad, San Bernardo, Santiago 8051919, Chile; elizz.belt@gmail.com; 10 CESFAM Raúl Cuevas, San Bernardo, Santiago 8071895, Chile; kmtapia@hotmail.com; 11 CESFAM Juan Pablo II, San Bernardo, Santiago 8053211, Chile; dani.mornhinweg@gmail.com; 12 Blanquerna School of Health Sciences, Global Research on Wellbeing (GRoW), Universitat Ramon Llull, 08025 Barcelona, Spain

**Keywords:** telerehabilitation, COVID-19, physical capacity, quality of life, fatigue, dyspnea

## Abstract

In many health systems, it is difficult to carry out traditional rehabilitation programs as the systems are stressed. We evaluate the effectiveness of a telerehabilitation program conducted in primary care in post-COVID-19 patients. An observational, prospective study was conducted in seven primary care centers in Chile. We included adult patients (>18 years) with a previous SARS-CoV-2 infection. The telerehabilitation program consisted of 24 sessions of supervised home-based exercise training. The efficacy was measured by the 1-min sit-to-stand test (1-min STST), the 36-Item Short Form Health Survey (SF-36), fatigue, and dyspnea symptoms before and after intervention. We included 115 patients (55.4% female) with a mean age of 55.6 ± 12.7 years. Fifty-seven patients (50%) had antecedents of hospitalization, and 35 (30.4%) were admitted to the ICU. The 1-min STST was improved after the intervention from 20.5 ± 10.2 (53.1 ± 25.0%predicted) to 29.4 ± 11.9 (78.2 ± 28.0%predicted) repetitions (*p* < 0.001). The SF-36 global score improved significantly from 39.6 ± 17.6 to 58.9 ± 20.5. Fatigue and dyspnea improved significantly after the intervention. Although limited by the absence of a control group, this report showed that a telerehabilitation program applied in primary health care is feasible and was effective in improving physical capacity, quality of life and symptoms in adult survivors of COVID-19.

## 1. Introduction

Post-Coronavirus disease 2019 (COVID-19) patients may experience persistent chest computed tomography (CT) abnormalities, decreased lung function, persistent fatigue and respiratory symptoms, decreased functional capacity and quality of life after symptom onset or hospital discharge [1,2].

Regarding functional capacity assessment in patients suffering from COVID-19 after the acute phase of the illness, it has become essential to estimate the magnitude of the disability and impairment of quality of life in the months following infection [3]. Initial reports have shown persistent impairments leading to functional consequences, at least at the timepoint of hospital discharge [4,5,6].

One non-pharmacological strategy that has traditionally been used in different chronic respiratory diseases is cardiopulmonary rehabilitation [7,8]. The first reports of this intervention in post-COVID-19 patients have shown to be effective in improving physical capacity and functional independence [9,10]. However, in many health systems, it is impossible to carry out traditional rehabilitation programs as the health systems are stressed, the infrastructure of physical medicine and rehabilitation services must be used in the care of acute patients, and the lower attendance of patients to hospital care for fear of being infected with COVID-19 [11] Additionally, many countries have reduced rehabilitation treatments for outpatients with chronic conditions in response to social distancing policies implemented to reduce the spread of the infection through the population [12].

From the aforementioned, the need arises to establish telerehabilitation programs so that people with post-COVID-19 sequelae can receive an intervention that helps minimize the functional impact of this disease on their lives [13]. Although there are only a few studies that have used telerehabilitation in patients with COVID-19 [14], this intervention has been shown to be effective in respiratory, cardiovascular, metabolic, and neurological pathologies [15].

Chile, a developing country, has an extensive network of primary care centers with rehabilitation professionals who until now have focused on the care of chronic respiratory patients, but due to the pandemic they have also absorbed patients with post-COVID-19 sequelae. Given that more complex hospitals are still treating acute patients, the alternative is to refer them to primary care. We aimed to evaluate the effectiveness of a telerehabilitation program conducted in primary care in post-COVID-19 patients.

## 2. Materials and Methods

### 2.1. Design and Setting

This was a multicentric, observational, and prospective study conducted in seven primary care centers in San Bernardo, Santiago, Chile. We followed the Strengthening the Reporting of Observational Studies in Epidemiology (STROBE) checklist [16]. This study was approved by the ethics committee (number 017-22032021) and all patients understood, agreed and signed the informed consent.

### 2.2. Participants

The participants were adult patients (>18 years) who had persistent dyspnea at post-discharge follow-up with a previous SARS-CoV-2 infection diagnosed by positive PCR assay findings for nasal and pharyngeal swab specimens, or according to chest computed tomography scan evidence [17]. We excluded patients with uncontrolled cardiovascular disease, those who saturated less than or equal to 93% with exercise, and cognitive impairment (based on medical criteria after the clinical assessment and the review of the patient’s medical history) [18].

### 2.3. Assessments

Candidate patients to enter the rehabilitation program were contacted by telephone for an appointment with the primary care center physician who conducted the interview, reviewed the medical history and decided if the patient met the inclusion criteria. If the patient did so, they were invited to participate and were assessed by the physiotherapist in person.

The following descriptive variables were assessed: socio-demographic data (age, sex), comorbidities, characterization of hospitalization (length of stay, days in mechanical ventilation). Physical capacity was assessed through the number of times that subjects were able to perform the 1-min sit-to-stand test (1-min STST) [19]. All tests were conducted within the presence of a trained physiotherapist. The test was performed using a chair of standard height (46 cm), without armrests, positioned against a wall. Participants were not allowed to use their hands/arms to push the seat of the chair or their body and were instructed to complete as many sit-and-stand cycles as possible in 60 s at self-paced speed [20]. The modified Borg scale (0–10) was used to measure dyspnea and fatigue immediately before and after the 1-min STST. A finger oximeter was used to record pulse oxygen saturation (SpO2) and heart rate (HR). We used the reference values based on the healthy adult population previously reported by Strassmann et al. [19].

The 36-Item Short Form Health Survey (SF-36) was used to measure health related quality of life (HRQoL) [21]. The score of each dimension is converted to a standard score ranging from 0 to 100, with the highest score indicating the best HRQoL [21]. Quality of life is evaluated according to eight different dimensions: Physical functioning (PF), Physical role functioning (RP), Bodily pain (BP), General health (GH), Vitality (VT), Social role functioning (SF), Emotional role functioning (RE), Mental health (MH).

Dyspnea symptoms were measured by the modified Medical Research Council (mMRC) dyspnea scale [22] which was used as a self-rating tool to measure the degree of disability that breathlessness poses on activities of daily living on a scale from 0 to 4. Participants were categorized as having dyspnea (mMRC 1–4) or no dyspnea (mMRC 0). Fatigue was assessed with a visual analogue scale between 0 and 10. Higher scores indicate a higher degree of fatigue [22].

### 2.4. Intervention

This program had a duration of nine weeks, carrying out 2 and 3 telerehabilitation sessions per week until the 24 sessions were completed. All patients were assessed in a primary care center at the beginning and at the end the program. Each telerehabilitation session was done at home. The program included warm-up (5 min), breathing exercises (3 min), aerobic and/or strength exercises (20–30 min), and stretching (5 min). The protocol was based on the recommendations of the American College of Sports Medicine [23] and the recommendations of respiratory physiotherapy and therapeutic exercise of the Colegio Profesional de Fisioterapeutas de la Comunidad de Madrid [24]. Weekly phone calls were made to evaluate user tracking. Depending on the patient’s condition, a program was conducted to moderate intensity—Borg scale ratings between 3 and 6. Physiotherapists taught patients to perform exercises with objects they had at home (such as chairs). Additionally, the program provides elastic bands adapted to each patient (Supplementary File S1). The program was performed by a physiotherapist from respective health centers, who carried out the initial and final evaluations and telephone follow-ups on a biweekly basis. The program was designed by the San Bernardo Health Direction. Then, the physiotherapists from each of the participating centers were trained. All physiotherapists had experience in the management of chronic respiratory diseases in regard to those who regularly perform cardiopulmonary rehabilitation. The telerehabilitation program was considered complete only in the subjects who performed 100% of sessions.

### 2.5. Statistics

All the analyses were performed using the statistical package SPSS version 25.0 software (IBM Corporation, Armonk, NY, USA). Quantitative variables were expressed as mean and standard deviation (SD) or median and interquartile range (IQR: 25th; 75th percentiles). Qualitative variables were described as frequencies and percentages (*n*, %). Baseline comparison between group characteristics was made using Fisher’s exact test, Student’s *t*-test or Mann–Whitney test (depending of the distribution) for qualitative or quantitative variables. A *p* < 0.05 was considered statistically significant.

## 3. Results

**Figure 1:**
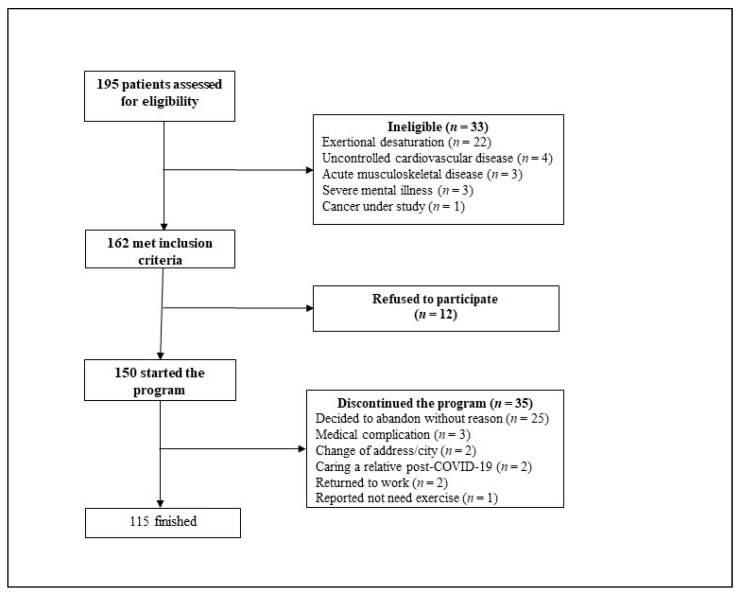
Flowchart of the selection process.

Primary care centers assessed 175 patients from 1 August 2020, until 15 February 2021. We excluded 13 patients (4 for uncontrolled cardiovascular disease, 3 for acute musculoskeletal pathology, 3 for severe mental illness, 2 for change of city, 1 for cancer under study). Of the patients suitable for telerehabilitation program, 162 met the inclusion criteria and were considered; 12 patients refused the program and were thus deemed ineligible. We started with 150 patients, but 35 abandoned the program (23%) (Figure 1). Finally, 115 patients (55.4% female) with a mean age of 55.6 ± 12.7 years completed the program. The duration between discharge of the acute COVID-19 phase and beginning of rehabilitation was 30 (27–35) days. In the baseline characterization of the patients, those hospitalized were mainly men and older, these differences being statistically significant. The most common comorbidities were hypertension (46%), obesity (40%), diabetes (25%), dyslipidemia (19%), arthrosis (15%), and asthma (13%). Fifty-seven patients (50%) had antecedents of hospitalization with a mean length of stay of 29.9 ± 19.9 days, and 35 (30.4%) were admitted to the ICU with a mean age of 19.6 ± 14.0 days on mechanical ventilation. The descriptive statistics are shown in Table 1.

The 1-min STST was improved after the intervention from 20.5 ± 10.2 (53.1 ± 25.0%predicted) to 29.4 ± 11.9 (78.2 ± 28.0%predicted) repetitions (*p* < 0.001). The patients with repetitions under the 2.5 percentile of reference value decreased from 51.3% to 15.7% (*p* < 0.001). The SF-36 global score improved significantly from 39.6 ± 17.6 to 58.9 ± 20.5, and this increase was observed in all dimensions of the questionnaire (Table 2). Fatigue and dyspnea symptoms improved significantly after the intervention.

In the analysis of non-hospitalized versus hospitalized patients, all outcomes improved with exception of fatigue in non-hospitalized patients (*p* = 0.065) and general health perception in hospitalized patients. All outcomes divided by group are shown in Table 2. In the analysis of ICU-admitted patients versus non-ICU-admitted patients all outcomes improved with the exception of four dimensions in the SF-36 (bodily pain, general health perceptions, emotional role limitations, mental health). All outcomes divided by ICU admission are shown in Table 3.

## 4. Discussion

A telerehabilitation program applied in primary health care effectively improved physical capacity, quality of life, and symptoms in adult survivors of COVID-19.

It is important to emphasize that our results align with what was observed in telerehabilitation programs for conditions such as osteoarthritis, low-back pain, hip and knee replacement, and multiple sclerosis and in the context of cardiac and pulmonary rehabilitation [15].

Physical capacity was evaluated with the 1-min STST, which has been recommended for telerehabilitation programs and is a good alternative when the 6-min walk test cannot be performed, mainly because it allows to maintain safe conditions when using a chair and has proven to be an excellent follow-up tool in face-to-face or remote rehabilitation programs [25,26,27,28]. In other pathologies, as chronic respiratory diseases, this test has been shown to have a good correlation with 6MWT [29,30]. Close to half of the patients showed a 1-min STST below the 2.5 percentile at the beginning of the program and only 15% at the end. Our results are in line with another telerehabilitation program in COVID-19 which shows that this test can be an alternative to evaluate the effectiveness of a rehabilitation program [31].

The minimal clinically important difference for 1-min STST in COVID-19 patients has not yet been reported. However, in COPD, the MCID for a rehabilitation intervention has been reported to be 2.5 repetitions (sensitivity: 80%, specificity: 60%) [32]. Although they are different diseases, it is important to emphasize that the change in our patients is four times that value.

This study was observational without a control group, for this reason it was difficult to determine how much of the improvement is due to the intervention itself and how much to the natural evolution of the disease. However, our results are in line with a previous study that applied a telerehabilitation program in 14 hospitalized patients (mean age 60.8 ± 10.4; 40% with ICU admission) [31]. The intervention group improve the 1-min STST by 10 points, and the control group improved 5 points. Additionally, 37% of the control group patients were still under the 2.5th percentile [31]. In our hospitalized patients, at the beginning, 65% of patients had a 1-min STST under the 2.5th percentile and post-intervention only 21% had lower than this value.

An important aspect to consider is that our study, like most of the studies that have been published on post-COVID-19 rehabilitation, does not have a control group [26]. However, the tests used have values with which they can be compared, such as the reference values of the sit-to-stand [19], and in the case of fatigue and dyspnea, it can be compared with respect to the absence of these symptoms. Undoubtedly, the lack of a control group is due to the unpredictability of the development of the pandemic, and in our case, because it is a service that our health system offers for all users, the design of the protocols must be improved to obtain more conclusive results.

Half of the studied population was hospitalized, and although significant changes in physical capacity were shown in the entire population, they were more significant in the hospitalized group. This may be because hospitalized patients, in addition to presenting the consequences of the virus, presented an average of one month of hospitalization, so the effects of prolonged rest are added [33]. In addition, most hospitalized patients were in the ICU for an average of almost 20 days, so we must add the effects of the drugs used in sedation [34]. It is important to consider that in the baseline characteristics, the hospitalized patients were mainly men and older, which was to be expected given that both are risk factors for severity and mortality [35]. Despite this, there were no differences between the groups in the baseline characteristics of the outcomes evaluated.

Quality of life and presence of symptoms were diminished after the intervention. All domains of SF-36 were significantly lower than the norm. Our results are in line with previous reports [36,37] and must be considered in the follow-up of functional limitations [34], particularly, as we have shown, because quality of life can be improved with rehabilitation.

The literature has shown that fatigue and dyspnea are two of the most prevalent symptoms in post-COVID-19 patients [38]. These findings have been closely related to the presence of long COVID-19 [39]. Therefore, therapeutic strategies such as telerehabilitation should be sought that can reduce the presence of symptoms, particularly in those who, in addition to persistent symptoms, have a significant deterioration in physical capacity and quality of life [13].

The possible mechanisms that support the realization of rehabilitation in post-COVID-19 patients are not yet fully elucidated. One of the possible explanations is that exercise improves neuromotor function, which would explain the improvement in physical tests in this population. Another possible explanation is that the functionality of most individuals was still severely restricted at the time-point of admission, particularly in subjects under mechanical ventilation [5,40]. These factors, added to the fact that the most severe patients have especially cardiometabolic comorbidities, cause the disease to have serious sequelae, and therefore, the potential for response to rehabilitation is greater.

The pandemic posed a challenge to carrying out the study thanks to recent advances in communication and technology, which allows the use of low-cost internet connections, smartphones and tablets, and the appearance of applications that facilitate video calls that have allowed the rapid adaptation of rehabilitation teams [41]. Before the pandemic, primary care in Chile and many other countries did not have telerehabilitation programs, which are now a reality and have proven to be effective. This study has highlighted the feasibility of a telerehabilitation program conducted in primary health care, even if the included patients presented very low functional exercise capacity and did not have specific equipment at home.

Dropout from our program was 23%, similar to that reported in rehabilitation programs for COPD patients in person, which vary between 10 and 32% [7]. Barriers, which prevent patients from attending rehabilitation programs include disruption of their everyday routine, inconvenient timing of the program, lack of perceived benefit, travel or transportation difficulties, lack of social support, low self-confidence, and fear of being breathless or exacerbating existing medical problems [7,42]. One might think that in the absence of transport barriers, adherence would be better, but the literature has shown that dropout rates are similar between face-to-face and tele-rehabilitation programs [43]. Although we must emphasize that the traditional way of calculating adherence is from the percentage of adherence of scheduled sessions in our case, we reported the loss rate as all subjects who abandoned the protocol since the patients had to complete the entire program to be considered finished.

Taking into consideration differences found in the outcomes of non-hospitalized versus hospitalized patients and ICU-admitted patients versus non-ICU admitted patients, it is necessary that future studies explore the specific effects of telerehabilitation in more severe cases, especially due to the literature reporting a high prevalence of lung function and functional impairment as well as substantial symptom burden in survivors of severe COVID-19 requiring mechanical ventilation [44].

Our study has some limitations. Our patients do not have a lung function study. Although some studies show pulmonary function tests at discharge, follow-up clinical guidelines suggest an evaluation at 2- or 3-months post-discharge [45,46]. A very early evaluation could show a more remarkable alteration due especially to the inflammation caused by the recent viral infection. On the other hand, we do not have a control group. This is because this study reports the clinical experience of moving from a face-to-face rehabilitation system that had to be quickly converted to telerehabilitation. In this sense, the literature has shown that telerehabilitation is similar to face-to-face rehabilitation and better than non-rehabilitation [15]. Additionally, this study reports the results of a “real-world” telerehabilitation program that is incorporated into the benefits received by primary care users who have post-COVID-19 sequelae in our city, so we did not design it with all the ideal conditions of a clinical trial such as having a control group. Finally, there could be a selection bias since the most severe patients, with chronic respiratory diseases and oxygen-dependent patients were referred to follow-up programs guided by teams of specialists in their referral hospitals.

## 5. Conclusions

Although limited by the absence of a control group, this report showed that our telerehabilitation program applied in primary health care is feasible and improved physical capacity, quality of life, and symptoms in adult survivors of COVID-19.

## Figures and Tables

**Table 1:** Patients’ characteristics.

Variable	All *n* = 115	Non-Hospitalized *n* = 58	Hospitalized *n* = 57
Gender (M/F)	49/66	14/44	35/22 *
Age, years	55.6 ± 12.7	51.6 ± 13.2	59.7 ± 10.8 *
Weight, kg	79.7 ± 15.8	81.3 ± 17.4	77.7 ± 13.4
Height, cm	155 ± 31	155 ± 29	155 ± 33
BMI	30.7 ± 5.3	31.5 ± 5.6	29.6 ± 46.5
Length of stay, days	-	0	29.9 ± 19.9
Requirement of MV, *n* (%)	35 (30.4)	0	35 (61.4)
Days on MV	-	0	19.6 ± 14.0
Comorbidities, *n* (%)			
Hypertension	53 (46.1)	21 (36.2)	32 (56.1)
Obesity	46 (40.0)	29 (50.0)	17 (29.8)
Diabetes	29 (25.2)	10 (17.2)	19 (33.3)
Dyslipidemia	22 (19.1)	9 (15.5)	13 (22.8)
Arthrosis	17 (14.8)	10 (17.2)	7 (12.3)
Asthma	15 (13.0)	14 (24.1)	1 (1.8)
Hypothyroidism	10 (8.7)	9 (15.5)	1 (1.8)
Congestive heart failure	4 (3.5)	2 (3.4)	2 (3.5)
Fibromyalgia	4 (3.5)	3 (5.2)	1 (1.8)
Chronic kidney disease	3 (2.6)	1 (1.7)	2 (3.5)
Arrhythmias	2 (1.7)	1 (1.7)	1 (1.8)
Depression	2 (1.7)	2 (3.4)	0 (0.0)
Deep venous thrombosis	1 (0.9)	0 (0.0)	1 (1.8)
Anemia	1 (0.9)	0 (0.0)	1 (1.8)
Alzheimer disease	1 (0.9)	0 (0.0)	1 (1.8)
Bronchiectasis	1 (0.9)	1 (1.7)	0 (0.0)
Pulmonary fibrosis	1 (0.9)	0 (0.0)	1 (1.8)
Liver damage	1 (0.9)	0 (0.0)	1 (1.8)

Abbreviations: BMI: body mass index; MV: mechanical ventilation. * *p* < 0.05 between hospitalized and non-hospitalized patients.

**Table 2:** Outcome measures results by hospitalization.

Variable	All Patients *n* = 115			Non-Hospitalized *n* = 58			Hospitalized *n* = 57			
	Pre	Post	*p*-Value	Pre	Post	*p*-Value	Pre	Post	*p*-Value	*p*-Value Intergroup *
Physical capacity, 1-min STST										
Repetitions	20.5 ± 10.2	29.4 ± 11.9	<0.001	24.2 ± 8.2	32.2 ± 12.6	<0.001	16.8 ± 10.8	26.5 ± 10.5	<0.001	0.008
%predicted	53.1 ± 25.0	78.2 ± 28.0	<0.001	61.7 ± 18.6	84.2 ± 29.6	<0.001	43.9 ± 27.4	71.9 ± 25.0	<0.001	0.021
Repetitions < 2.5th perc, *n* (%)	59 (51.3)	18 (15.7)	<0.001	22 (37.9)	6 (10.3)	<0.001	37 (64.9)	12 (21.1)	<0.001	<0.001
Quality of life, SF-36										
Physical functioning	44.7 ± 28.4	67.2 ± 26.0	<0.001	53.0 ± 24.4	71.9 ± 24.6	<0.001	35.6 ± 29.8	62.0 ± 26.7	<0.001	0.046
Physical role limitations	12.9 ± 29.6	47.1 ± 43.5	<0.001	18.3 ± 34.5	56.9 ± 44.2	<0.001	6.9 ± 21.8	36.3 ± 40.4	<0.001	0.012
Bodily pain	47.9 ± 27.7	60.4 ± 27.7	<0.001	48.3 ± 25.2	61.4 ± 26.3	<0.001	47.3 ± 30.5	59.2 ± 29.4	0.010	0.790
General health perceptions	49.2 ± 18.8	55.0 ± 19.9	0.007	48.1 ± 17.5	54.9 ± 18.0	0.001	50.3 ± 20.3	55.1 ± 22.0	0.154	0.984
Energy/vitality	40.7 ± 21.7	58.5 ± 21.2	<0.001	42.9 ± 20.5	58.7 ± 22.7	0.017	38.3 ± 22.9	58.3 ± 19.7	<0.001	0.912
Social functioning	46.6 ± 30.0	71.2 ± 30.6	<0.001	50.6 ± 27.9	75.9 ± 26.8	<0.001	42.3 ± 31.8	66.1 ± 33.8	<0.001	0.112
Emotional role limitations	30.4 ± 40.8	54.9 ± 43.2	<0.001	32.9 ± 43.2	62.5 ± 41.0	<0.001	27.7 ± 38.2	46.5 ± 44.3	0.006	0.041
Mental health	51.0 ± 25.2	63.7 ± 24.9	<0.001	49.9 ± 22.7	65.5 ± 23.6	<0.001	52.2 ± 27.9	61.8 ± 26.4	0.010	0.451
Total score	39.6 ± 17.6	58.9 ± 20.5	<0.001	41.9 ± 16.8	63.3 ± 19.4	<0.001	37.1 ± 18.2	54.1 ± 20.8	<0.001	0.038
Symptoms										
Dyspnea, mMRC score	2 (1–3)	1 (0–2)	<0.001	2 (1–3)	1 (0–1)	<0.001	2 (2–3)	1 (0–2)	<0.001	0.031
Fatigue, VAS	3 (0–5)	1 (0–3)	<0.001	1.5 (0–4)	1 (0–2)	0.065	3 (1–5)	1 (0–3)	0.001	0.448

Abbreviations: 1-min STST: 1-min sit-to-stand test; mMRC: Modified Medical Research council; VAS: visual analogue scale. * Comparison of post-intervention results between non-hospitalized vs. hospitalized patients. Intra and intergroup comparison pre and post-intervention using paired t-test for continuous variables and the Mann–Whitney U test if the distribution was skewed.

**Table 3:** Outcome measures results by ICU admission.

Variable	Non-ICU *n* = 21			ICU *n* = 36			
	Pre	Post	*p*-Value	Pre	Post	*p*-Value	*p*-Value Intergroup *
Physical capacity, 1-min STST							
Repetitions	18.9 ± 10.4	29.2 ± 10.7	<0.001	15.1 ± 10.9	25.1 ± 10.2	<0.001	0.271
%predicted	49.9 ± 27.7	76.2 ± 28.5	<0.001	39.4 ± 26.4	69.5 ± 22.9	<0.001	0.405
Repetitions < 2.5th perc, *n* (%)	12 (57.1)	3 (14.3)	<0.001	25 (69.4)	9 (25.0)	<0.001	0.435
Quality of life, SF-36							
Physical functioning	41.7 ± 28.9	67.9 ± 22.4	<0.001	31.8 ± 29.7	58.6 ± 28.6	<0.001	0.339
Physical role limitations	13.8 ± 31.9	42.1 ± 41.7	0.019	2.9 ± 10.1	32.6 ± 39.3	<0.001	0.590
Bodily pain	51.3 ± 32.6	74.2 ± 26.0	0.008	44.9 ± 28.8	51.8 ± 28.8	0.212	0.013
General health perceptions	51.5 ± 19.1	62.6 ± 22.4	0.009	48.9 ± 20.9	50.3 ± 20.6	0.842	0.087
Energy/vitality	44.3 ± 27.1	62.4 ± 20.7	<0.001	37.6 ± 21.6	55.9 ± 18.7	<0.001	0.385
Social functioning	45.3 ± 31.5	76.7 ± 30.9	<0.001	42.0 ± 32.2	60.7 ± 34.1	0.006	0.136
Emotional role limitations	34.1 ± 40.6	63.4 ± 42.9	0.003	26.7 ± 39.4	35.3 ± 42.5	0.224	0.032
Mental health	57.8 ± 29.2	71.7 ± 20.7	0.004	49.2 ± 27.5	56.2 ± 27.6	0.220	0.058
Total score	42.1 ± 19.5	63.7 ± 18.6	<0.001	34.9 ± 16.9	48.8 ± 19.8	<0.001	0.019
Symptoms							
Dyspnea, mMRC score	2 (2–3)	1 (0–2)	<0.001	3 (2–3)	1 (0–2)	0.002	0.648
Fatigue, VAS	3 (0–4)	1 (0–3.25)	0.053	3 (1.75–5)	1.5 (0–2.75)	0.004	0.473

Abbreviations: 1-min STST: 1-min sit-to-stand test; ICU: intensive care unit; mMRC: Modified Medical Research council; VAS: visual analogue scale. * Comparison of post-intervention results between non-hospitalized vs. hospitalized patients. Intra and intergroup comparison pre and post-intervention using paired t-test for continuous variables and the Mann–Whitney U test if the distribution was skewed.

## Data Availability

The data supporting the findings of this study are available from the corresponding author upon request.

## Supplementary Materials

The following are available online at https://www.mdpi.com/article/10.3390/jcm10194428/s1, File S1. Exercise guide for patients.
