# Supplementary material for: Effectiveness of a Primary Care Telerehabilitation Program for Post-COVID-19 Patients: A Feasibility Study
Source: J Clin Med. 2021 Sep 27;10(19):4428. doi: 10.3390/jcm10194428 (PMC8509356; doi:10.3390/jcm10194428)
Supplement: Supplementary file 1 [file jcm-10-04428-s001.zip › jcm-1321459-SI.pdf]

Remote rehabilitation program for COVID-19 patients

Corporación Municipal de Salud y Educación San Bernardo.

Dirección de Salud.

August 2020.

Version 1

Based on the recommendations of respiratory physiotherapy and therapeutic exercise for people confined at home and/or in the home recovery phase of COVID-19. Colegio Profesional de Fisioterapeutas Comunidad de Madrid. https://cfisiomad.org

This document corresponds to the Manual of the Cardiopulmonary Rehabilitation Program for primary care patients of the Commune of San Bernardo.

You are a user who has suffered from a disease known as Coronavirus or COVID-19. This disease affects not only the lungs but also the entire body. Given that you had to rest or were hospitalized, it is most likely that with this lack of mobility, added to the preventive isolations decreed by the health authority, you may present a decrease in muscle strength and cardiopulmonary capacity. Therefore, it is important to perform physical exercise daily, and in particular, the purpose of this program is to perform moderate to high-intensity training 2 to 3 times a week.

The duration is nine weeks, the first two weeks carrying out two intervention sessions and the following seven weeks three sessions to complete 24 sessions. Considering that the quarantines can be extended, the program is designed for you to carry out in its entirety at your home.

We recommend accompanying this training with a good diet and not stopping any drug used for chronic diseases. You must notify the follow-up team if new symptoms appear at any time during the procedure.

It is important to note that you will need to record your efforts on the program session sheet at the end of this manual.

MANAGEMENT OF SHORTNESS OF BREATH

One of the symptoms that persist after a COVID hospitalization is shortness of breath. This can lead to feelings of anxiety which can increase shortness of breath. Staying calm is the best way to manage shortness of breath, along with the following tips.

Shortness of breath should increase as the exercises in the program are performed but should not be limiting of performance.

If the shortness of breath is very intense, it is recommended to adopt the following positions.

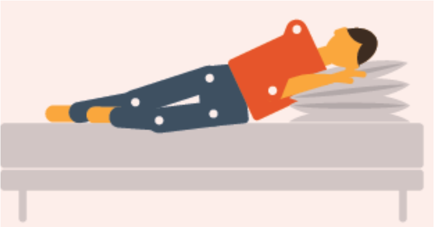

1.- Lying on its side.

Lying on one side with abundant pillows or cushions, supporting head and neck, with slightly bent knees.

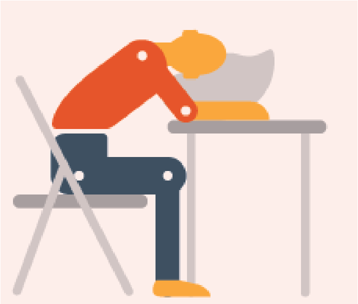

2.- Sitting with anterior tilt.

Sitting in front of a table, leaning forward from the waist with head and neck resting or not on a cushion or pillow, and arms resting on the table.

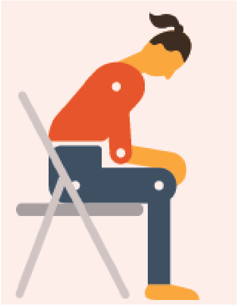

3.- Sitting with anterior inclination (without table in front).

Sitting on a chair, leaning the arms forward supported on the thighs or on the armrests of the chair.

WARM UP

Warm-up exercises prepare the body for exercise and prevent injury. This warm-up should last 3 to 5 minutes, and at the end you should feel a slight shortness of breath. It is recommended to perform the warm-up series in 3 series of 10 repetitions and the elongations to maintain for at least 10 seconds.

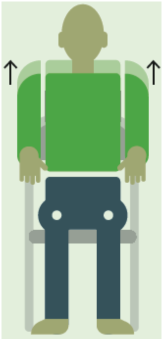

1.- Shoulder raise.

Slowly raise the shoulders towards the ears and then return to the starting position.

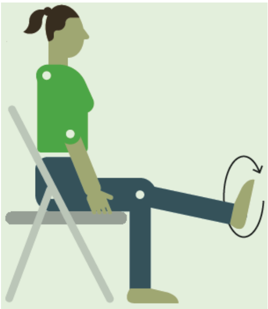

2.- Ankle circles.

Using one foot, draw circles with your toes, repeat with the other foot.

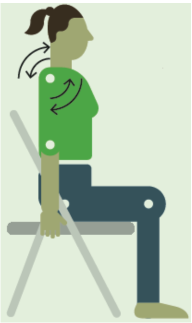

3.- Shoulder circles.

Keeping your hands resting on the chair or legs, slowly move your shoulder around in a forward and backward circle.

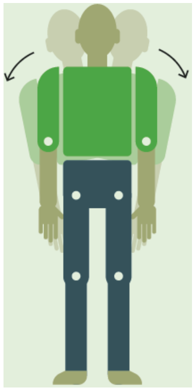

4.- Inclinations.

Start with your body in a neutral position. You can do it standing or sitting. Approach one arm towards the ground and then the same with the other side.

You can also perform only neck bends.

BREATHING EXERCISES.

Breathing exercises aim to improve lung function and the ventilatory pattern, which can be affected by diseases such as COVID-19, especially if it is accompanied by Pneumonia. These are exercises that seek to increase the volume of inspired air and reduce work of breathing or difficulty breathing.

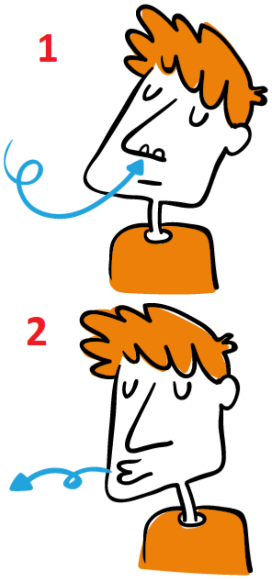

1.- Pursed-lip breathing.

Performing this exercise can help reduce feelings of shortness of breath. We can use it accompanied by arm exercises.

Breathe in slowly through the nose until reaching our maximum capacity.

Hold 2-3 seconds if you can.

Blowing slowly through the mouth forming a U with the lips, as if we were blowing the air through a light bulb.

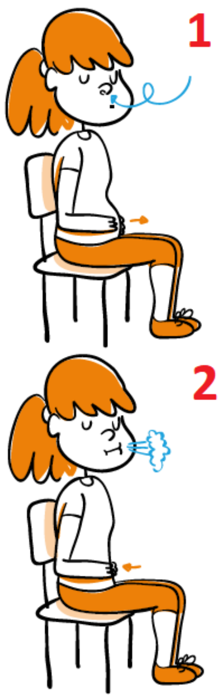

2.- Abdominal or diaphragmatic breathing.

Starting position sitting in a chair with your hands on your abdomen.

From this position, notice how the abdomen inflates when you take in air (inhale) and hides when you take the air out (exhale).

Take air through the nose (as much as possible) and slowly exhale it through the mouth with pursed lips.

Perform not only before exercise, but during the morning and afternoon.

Repeat for at least 10 breaths.

If you have a cough with expectoration, the following exercises are recommended. Their objective is to improve the transport of secretions at the pulmonary level. They can be used in the morning and the afternoon. During these techniques, it is important to be in a well-ventilated space, and you must observe protection and hygiene measures with the management of your secretions.

(Based on the recommendations of respiratory physiotherapy and therapeutic exercise for people confined at home and/or in the home recovery phase of COVID-19. Colegio Profesional de Fisioterapeutas Comunidad de Madrid. https://cfisiomad.org)

Expiration with the aid of domestic positive expiratory pressure:

This exercise will help you to move secretions that may be deep in the lungs towards the area near the mouth and then we will expel with a cough. We will make exhalations in a bottle of water through a bulb or plastic tube for about 3 minutes.

Slow expiration exercises with an open mouth:

This exercise will help move secretions that may be "deeper" in the lungs towards the area closest to the mouth, and then we will expel with a cough.

Lying on your side on a flat surface (such as a bed) or sitting in a chair.

Breathe in through the nose in a normal way.

Breathe out slowly with your mouth open until all the air is drained from your lungs.

Perform for 3 minutes, and repeat the same lying on the other side.

Perform twice a day (morning and afternoon).

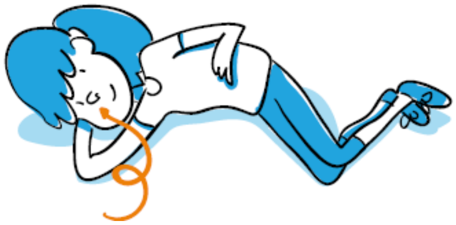
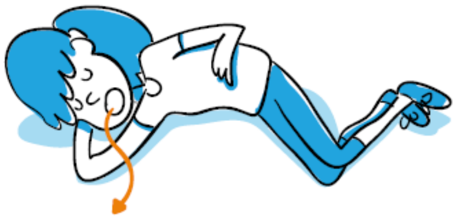

AEROBIC/STRENGTH EXERCISES.

Aerobic/Strength exercises should last 20 to 30 minutes at an initial stage to advance to 30 to 45 minutes at the end of the program with an interval training modality (moderate to high-intensity sets followed by a rest period). Any activity that causes moderate to almost severe shortness of breath is considered suitable for improving strength. Therefore, the recommendation is to do the most repetitions in 1 minute and rest for 1 minute. If it cannot be calculated in this way, we recommend starting with series of 10 repetitions and gradually increasing the number of repetitions in such a way as to perceive shortness of breath appropriate to the intensity.

(Based on the recommendations of respiratory physiotherapy and therapeutic exercise for people confined at home and/or in the home recovery phase of COVID-19. Colegio Profesional de Fisioterapeutas Comunidad de Madrid. https://cfisiomad.org)

How do I know how much intensity I need to do so that exercise is adequate?

The intensity of the strength exercises will be graded with a BORG scale for DYSPNEA (shortness of breath) and FATIGUE (lack of strength in the legs or arms).

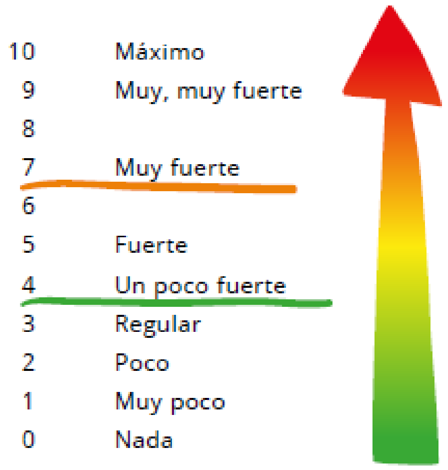

(Based on the recommendations of respiratory physiotherapy and therapeutic exercise for people confined at home and/or in the home recovery phase of COVID-19. Colegio Profesional de Fisioterapeutas Comunidad de Madrid. https://cfisiomad.org)

To know if the intensity of exercise is adequate, take into account the following:

- If you can say a complete sentence without stopping or shortness of breath, the intensity is very low.
- If you cannot speak at all or can only say a word while doing activity accompanied by dyspnea, the intensity of exercise is very high.
- If you can say a phrase, stopping once or twice for air and your shortness of breath is moderate to almost severe, the training is at the right intensity.

IF DURING EXERCISE I HAVE TO BREATHE FOR ONE OR TWO TIMES WHEN SAYING YOUR FULL NAME, THE INTENSITY IS ADEQUATE.

LOWER EXTREMITIES

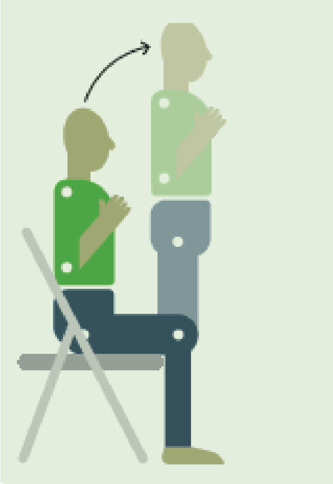

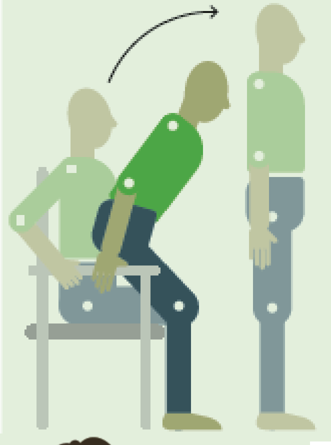

1.- Sit to stand.

Sit in a chair with your feet in the same line as your hips (not feet together). Arms can be at the side or crossed on the chest, stand up slowly holding the position, and sit SLOWLY. Keep your feet flat on the floor at all times.

If it is not possible to do this activity without using your arms, try a higher chair. If this is still impossible, use a chair with armrests to facilitate movement.

Intensity progression:

Perform more repetitions.

Change the chair for a lower one.

Carry a weight in your hands for activity.

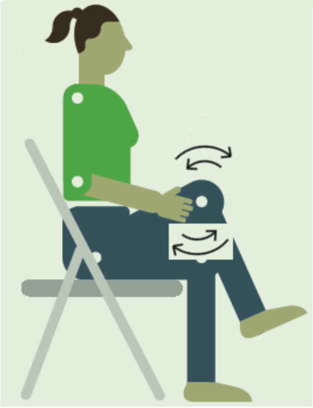

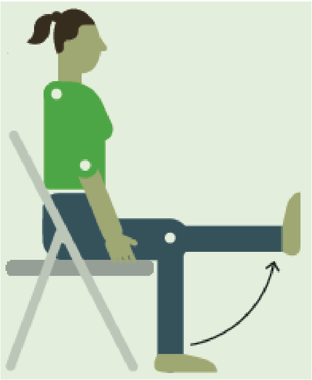

2.- Imaginary bycicle

Sitting in a chair. Perform the movement interspersed, resembling pedaling on an imaginary bicycle. For this, you must support your arms so as not to lose balance and your feet should not touch the ground while performing the repetitions.

If it is not possible to carry out this activity, modify the lower image exercise. Sitting with your feet together, stretch one knee forward and slowly bring yourself back to the starting position in order to control movement when returning. Repeat the same with the other leg.

Intensity progression:

Perform more repetitions.

Use elastic band between chair and ankle.

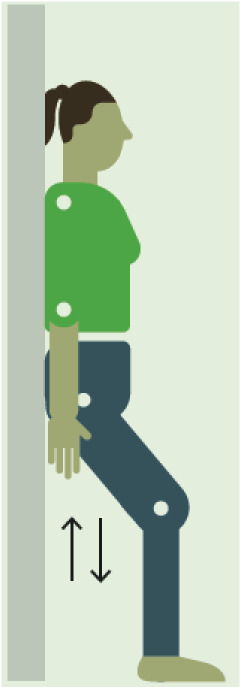

3.- Squat.

Stand with your back against a wall with your ankles about a foot away from the wall. Hands should be at the side. If you can't hold the position, lean forward with a chair.

Keeping your back against the wall at all times, slowly slide down the wall until you reach 90 ° of knee flexion. The knees should not be in front of the ankles when going down.

Then slowly work your way up to the starting position.

Intensity progression:

Perform a greater number of repetitions.

Load weight on your hands while doing the exercise.

UPPER EXTREMITIES.

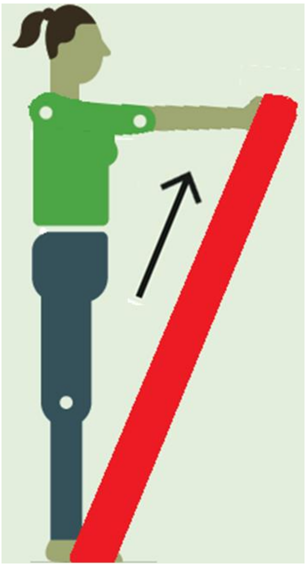

1.- Shoulder flexion.

With your arms at your side, holding a rubber band or weight in each hand and with your elbow extended.

From this position, raise your hand keeping the elbow extended forward.

Return to the starting position, controlling the movement.

Intensity progression.

Increase resistance as you do the shoulder flexion.

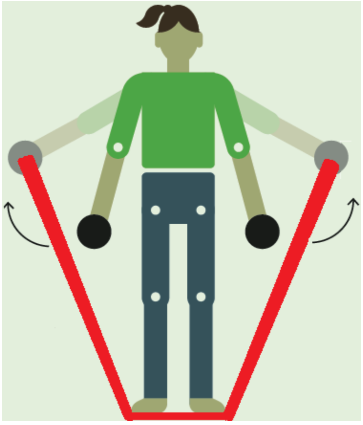

2.- Arms elevation.

Hold a weight or elastic band in both hands with your arms at your side.

Raise both arms to the side with elbows extended and then return to the starting position.

When lifting you should try to get as high as possible, ideally at shoulder height.

Intensity progression.

Increase resistance as you do the shoulder lift.

Increase lift height.

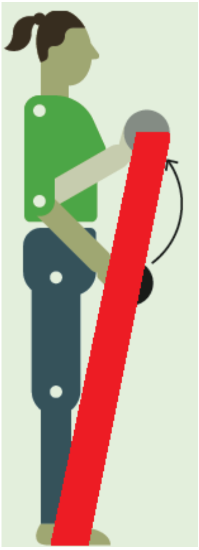

Elbow flexion.

With your arms at your side, holding a rubber band or weight in each hand.

Keep the elbow close to the body. Raise the most distal end of the arm (forearm) bringing the hand closer to the shoulder on the same side. Then return to starting position.

You can perform this exercise standing or sitting.

Intensity progression.

Increase the resistance with which the elbow flexion is performed.

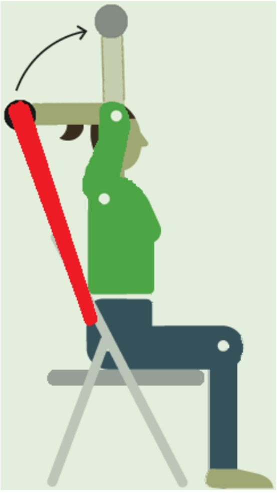

4.- Elbow extension.

With one of your arms above shoulder level and elbow at 90 ° and holding one end of the elastic band. The other arm holds the other end of the elastic band from behind.

Keep the elbow close to the face. Raise the most distal end of the arm (forearm) by bringing the hand back and forth and upward in order to stretch the arm upward. If there is no elastic band, it can be replaced with some weight and the direction of movement is the same.

Intensity progression.

Increase the resistance with which the elbow extension is performed.

The strength training modality is interval, so it is suggested to perform cycles of 1 minute of activity at 80% of maximum capacity (the maximum number of repetitions in a unit of time) for 1 minute of rest. An alternative way is by counting the number of repetitions performed for each cycle. It is recommended to perform at least three sets of each exercise before moving on to the next. The way to quantify the intensity is with the BORG scale and/or with dyspnea self-evaluation when speaking.

To increase the intensity, the number of repetitions performed per cycle must be increased.

For each strength exercise (Arms and leg) the following scheme should be followed.

alta intensidad		15 repeticiones		15 repeticiones		15 repeticiones	
reposo			reposo		reposo		reposo
		1 minuto	1 minuto	1 minuto	1 minuto	1 minuto	1 minuto
